# The Use of a Mercury Biosensor to Evaluate the Bioavailability of Mercury-Thiol Complexes and Mechanisms of Mercury Uptake in Bacteria

**DOI:** 10.1371/journal.pone.0138333

**Published:** 2015-09-15

**Authors:** Udonna Ndu, Tamar Barkay, Robert P. Mason, Amina Traore Schartup, Radwan Al-Farawati, Jie Liu, John R. Reinfelder

**Affiliations:** 1 Department of Environmental Sciences, Rutgers University, New Brunswick, New Jersey, United States of America; 2 Department of Biochemistry and Microbiology, Rutgers University, New Brunswick, New Jersey, United States of America; 3 Department of Marine Sciences, University of Connecticut, Groton, Connecticut, United States of America; 4 Department of Environmental Health, Harvard School of Public Health, Boston, Massachusetts, United States of America; 5 Marine Chemistry Department, King Abdul-Aziz University, Jeddah, Saudi Arabia; Cornell University, UNITED STATES

## Abstract

As mercury (Hg) biosensors are sensitive to only intracellular Hg, they are useful in the investigation of Hg uptake mechanisms and the effects of speciation on Hg bioavailability to microbes. In this study, bacterial biosensors were used to evaluate the roles that several transporters such as the glutathione, cystine/cysteine, and Mer transporters play in the uptake of Hg from Hg-thiol complexes by comparing uptake rates in strains with functioning transport systems to strains where these transporters had been knocked out by deletion of key genes. The Hg uptake into the biosensors was quantified based on the intracellular conversion of inorganic mercury (Hg(II)) to elemental mercury (Hg(0)) by the enzyme MerA. It was found that uptake of Hg from Hg-cysteine (Hg(CYS)_2_) and Hg-glutathione (Hg(GSH)_2_) complexes occurred at the same rate as that of inorganic complexes of Hg(II) into *Escherichia coli* strains with and without intact Mer transport systems. However, higher rates of Hg uptake were observed in the strain with a functioning Mer transport system. These results demonstrate that thiol-bound Hg is bioavailable to *E*. *coli* and that this bioavailability is higher in Hg-resistant bacteria with a complete Mer system than in non-resistant strains. No difference in the uptake rate of Hg from Hg(GSH)_2_ was observed in *E*. *coli* strains with or without functioning glutathione transport systems. There was also no difference in uptake rates between a wildtype *Bacillus subtilis* strain with a functioning cystine/cysteine transport system, and a mutant strain where this transport system had been knocked out. These results cast doubt on the viability of the hypothesis that the entire Hg-thiol complex is taken up into the cell by a thiol transporter. It is more likely that the Hg in the Hg-thiol complex is transferred to a transport protein on the cell membrane and is subsequently internalized.

## Introduction

Several biosensors have been developed for the detection and quantification of inorganic mercury (Hg(II)) in bacterial cells [1–6]. These tools have been used to assess the bioavailability and uptake pathways of Hg(II) complexes to microorganisms as they have the advantage that they only respond to Hg that has entered the cell [2–6]. These bioassays may therefore be applicable to the evaluation of effects of ligand complexation on Hg(II) bioavailability, the deciphering of mechanisms of Hg(II) uptake, and the involvement of various transport pathways in the uptake of Hg(II). With the use of biosensors it has been shown that formation of neutral inorganic mercury complexes such as HgCl_2_ results in increased uptake while charged complexes (HgCl_3_^-^, HgCl_4_^2-^) cause a reduction of Hg(II) uptake [7], results that are similar to those obtained using other approaches [8,9].

Using an *E*. *coli mer*-*lux* biosensor, it was shown [10] that the bioavailability of Hg(II) decreased as the concentration of thiosulfate increased, and that Hg(S_2_O_3_)_2_^2-^ was more bioavailable than Hg(S_2_O_3_)_4_^3-^. Other studies using biosensors have demonstrated that increased binding of Hg(II) and methylmercury (MeHg) with natural dissolved organic matter, such as humic acids, resulted in decreased uptake of both Hg(II) and MeHg [11]. Biosensors have also been used to demonstrate the effect of cellular membranes on the uptake of Hg(II) [12]. The effect of thiol complexation on the bioavailability of Hg(II) and MeHg was examined with the use of biosensors in a recent study [11] which reported that binding of Hg(II) and MeHg with the thiol cysteine increased the bioavailability of Hg(II) and MeHg while mercury-glutathione complexes (Hg(GSH)_2_, MeHg-GSH) [11,13] were not as bioavailable as neutral complexes or mercury-cysteine complexes (Hg(CYS)_2_, MeHg-CYS). This result is consistent with previous studies [13,14] that had used a cell surface wash to remove surface-bound Hg so that the measured Hg was considered equivalent to the Hg within the cells. This approach was used to evaluate Hg(II) uptake and show that complexation with cysteine increases the uptake and methylation of Hg, supporting the results from the bioreporter studies.

Whereas several studies have shown that mercury-thiol complexes are bioavailable [11,13,14] there are few studies detailing the mechanisms of Hg(II) uptake, and the role various transporters play in the uptake process. Passive diffusion of Hg(CYS)_2_ and Hg(GSH)_2_ across cell membranes is unlikely given that these complexes are negatively charged at environmental pH values, with the presence of carboxylic and amine groups on the thiolated compounds making them polar. The complexes are also fairly large which would hinder passive uptake. Two models have been proposed to explain the bioavailability of Hg in mercury-thiol complexes: the thiol-transporter model [13,15], and the membrane-exchange model [14]. The thiol-transporter model postulates that thiol transporters, which generally transport thiols into cells, can also bring mercury-thiol complexes into a cell [13]. The membrane exchange hypothesis proposes that there is an exchange of Hg from the complex to a transport protein on the membrane surface of the cell, followed by internalization of the Hg [14]. To further examine the relative importance of these pathways in bacteria, we examined the effect of the presence of the glutathione transport system in *E*. *coli* (14) on uptake as well as the influence of the cystine symporter system in *Bacillus* on Hg(II) uptake. Well-described microbial thiol transporter systems exist and can be used to test the validity of the thiol transporter model. In contrast, we used bacteria with the Mer transporters as a model system to test the second hypothesis as it is known that the Mer system involves a cell surface transporter for Hg(II) uptake.

It was previously established that uptake of glutathione in *Escherichia coli* is catalyzed by an ATP-binding cassette family (ABC) of transporters which include γliA, γliB, γliC, and γliD [16]. Suzuki *et al*. [16] described the glutathione importer as a system consisting of 4 proteins (γliABCD) where the structures γliA and —B are an ATP binding domain and perisplasmic binding protein, respectively, and γliC and —D are integral membrane proteins. Hence, we postulated, based on the thiol-transporter hypothesis, that this transport system would also be capable of importing mercury-glutathione complexes into a cell.

Cysteine transport in microorganisms is not well understood. Although two kinetically distinct transport systems of cysteine/cystine (a general transport system, *K*_*m*_ = 3 × 10^−7^ M; and specific transport system, *K*_*m*_ = 2 ×10^−8^ M) have been recognized in *E*. *coli* [17], the genes that encode these transporters have not been identified to date. In the gram positive bacterium *Bacillus subtilis* 168, three different transport systems for cystine including 2 ATP binding cassette transporters (YtmJKLMN and YckKJI), and a symporter (YhcL) have been identified [18]. Of these, the symporter was shown to be the primary importer of cystine into the cell. Burguiere et al. [18] also demonstrated that several analogues of cystine such as cysteine, seleno-DL-cystine, and *S*-methyl-L-cysteine competitively inhibited the uptake of cystine through the symporter YhcL indicating that on some level this protein also exhibited transport capabilities for these analogues. Given the structural similarity of Hg(CYS)_2_ and cystine, we tested the hypothesis that this transport system is also capable of transporting Hg(CYS)_2_ by comparing uptake rates in the wildtype *B*. *subtilis* 168 which has a YhcL functioning transport system to mutant strains where this transport function had been disrupted by knockout of the gene coding for the cysteine/cystine symporter.

The Mer transporters MerE, and MerT along with the periplasmic protein MerP are found in many Hg-resistant bacteria and have been shown to be effective transporters of Hg(II) [19–21] and MeHg [22] into the cells where they are transformed by the Mer system (MerA and/or MerB). However, to date there is little information on the bioavailability of thiol-bound Hg to these Hg-resistant bacteria, and what roles the Mer transport system play in the uptake of Hg from such complexes. Therefore, the objectives of this study were to examine the bioavailability of cysteine and glutathione complexes of Hg(II) to Hg-resistant strains of *E*. *coli*, and to probe the roles of glutathione, cysteine/cystine, and Mer transporters in the uptake of Hg when present as these complexes in the medium. In this study, we used a novel biosensor approach where the amount of intracellular conversion of Hg(II) to elemental mercury (Hg(0)) by the enzyme MerA is used as a surrogate for uptake. The assumption, as discussed further below, is that only Hg(II) transported into the cell is converted to Hg(0) and that other pathways of reduction are insignificant compared to this pathway. We show that this is the case in the studies reported here, and that the measure of Hg(0) in the medium is a true reflection of the relative uptake of Hg when bound as different complexes in the medium.

## Materials and Methods

### Materials

The mercury standard used in this study was obtained from Brooks Rand Corporation. The growth medium used was Luria Bertani (LB) while the medium used in mercury uptake assays was M9 [23], a defined medium. The use of a defined medium was necessary to control mercury speciation. M9 medium includes Na_2_HPO_4_ (42 mM), KH_2_PO_4_ (22 mM), NaCl (9 mM), MgSO_4_ (1 mM), NH_4_Cl (18.7 mM)), and glycerol (0.4%). Yeast and vitamins typically added to this medium were excluded. Acid washed 40 mL borosilicate glass clear vials were used for all assays. Mercury analysis was performed with the Hydra AA Automated Mercury Analysis System. The antibiotics used in the growth medium were ampicillin (100 μg mL^-1^) or tetracycline (30 μg mL^-1^).

### *E*. *coli* Strains

**Fig 1:**
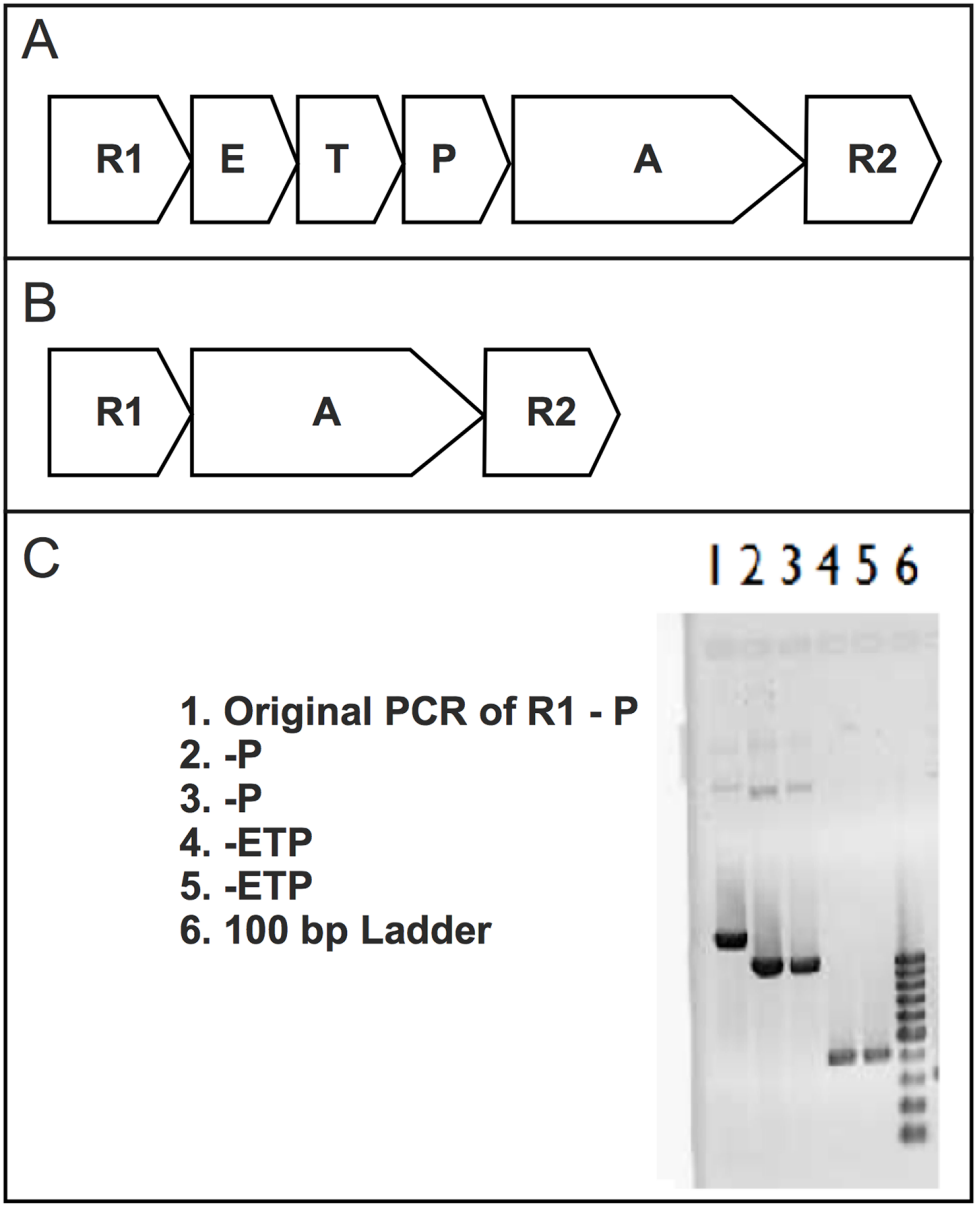
Diagrams of plasmids used in this study and a gel showing modification of plasmids. A. The *mer* operon of plasmid pHYRIA. B. The plasmid pHYRIAX is a modification of pHYRIA by the deletion of *E*, *T*, and *P*. C. Gel showing deletion of the genes *E*, *T*, & P from the plasmid pHYRIA.

The *E*. *coli* strains used in this study were MER, NRBZ1, and SINM35 (Table 1). The MER strain was obtained from the study of Chien et al. [24] and contains a mercury resistant plasmid (pHYRIA, Fig 1A) which has the *mer* genes *A*, *T*, *P*, *E*, *R* and the *mer* promoter region from *Bacillus megaterium*. In order to study the role of Mer transport proteins, the plasmid pHYRIA was modified by the deletion of the genes *E*, *T*, and *P* using the Quikchange® Site-Directed mutagenesis kit (Agilent Technology). Gene deletions were achieved by the use of a polymerase chain reaction with specially designed primers (Table 2) that excluded the targeted genes (*E*, *T*, and *P*) from amplification by PfuTurbo DNA polymerase (a high fidelity DNA polymerase). PCR cycling conditions are provided in Table 3. PCR was followed by a 1 hour digest with *Dpn*I which degrades template DNA, but not PCR products. The modified plasmid (pHYRIAX, Fig 1B) was transformed into *E*. *coli* strain XL gold cells (now designated as NRBZ1) as described in the manufacturer’s manual and spread on plates containing 100 μg mL^-1^ of ampicillin. Sequencing of the plasmid pHYRIAX was performed, and the results indicate that the genes *E*, *T*, and *P* were deleted. Colony PCRs (using primers D1 and D2), show the modification of the plasmid pHYRIA in Fig 1C. The plasmid pHYRIAX was also transformed into the *E*. *coli* strain SI35 and designated SINM35. Selection of transformants was by use of ampicillin (100 ug mL^-1^). SI35 is a mutant of the *E*. *coli* strain K12 where genes γ*liA* and γ*liB* that encode for part of the glutathione ATP-binding cassette transport system, and the gene *γ-glutamyltranspeptidase* (*ggt*), which encodes an enzyme that catalyzes the breakdown of glutathione in the periplasm were deleted from the chromosome [16]. As the genes coding the Mer transport system (*E*, *T*, and *P*) were deleted in pHYRIAX, uptake of Hg(II) into strains NRBZ1 and SINM35 cannot be attributed to the Mer Transport system.

**Table 1:** A list of strains used in this study.

Strain	Description	Source
*Escherichia coli* (MER strain)	pHYRIA + pET-MB1B1; contains MerA, MerE, MerT, MerP	[24]
*Escherichia*. *coli* NRBZ1	pHYRIAX	This study
*Escherichia coli* SI35	Δ*yliAB*; glutathione transport knockout	[16]
*Escherichia coli* SINM35	*E*. *coli* SI35 + pHYRIAX	This study
*Bacillus subtilis* 168		[18]
*Bacillus subtilis* 1534	ΔYhcL; cystine symporter knockout	[18]
*Bacillus subtilis* 1389	Δ*ytmJKLMN*::*aphA3*	[18]
*Bacillus subtilis* 168X	*B*. *subtilis* 168 + pHYRIAX	This study
*Bacillus subtilis* 1534X	*B*. *subtilis* 1534 + pHYRIAX	This study
*Bacillus subtilis* 1389X	*B*. *subtilis* 1389 + pHYRIAX	This study

**Table 2:** Primers used in this study.

Primer Name	Sequence 5' to 3'	Amplified Regions
ETPf	TCAGGTTGGTTTCTTGTCGCAATTAAGACGGAGGTTTC	All of pHYRIA except sections of *E*, *T* and sections of *P*
ETPr	GAAACCTCCGTCTTAATTGCGACAAGAAACCAACCTGA	All of pHYRIA except sections of *E*, *T* and sections of *P*
D1	CAGAGCCTGAACGTACGGAA	
D2	TTATTCGACGGGGAGACCCA	

**Table 3:** PCR Cycling Parameters.

	No of Cycles	Temperature °C	Duration
1	1	95	1 min
2	18	95	50 sec
		60	50 sec
		68	1 min/kb
3	1	68	7 minutes

### *Bacillus* Strains

The *Bacillus* strains, wildtype *B*. *subtilis* 168, and the mutants *B*. *subtilis* 1534 and *B*. *subtilis* 1389 were provided by I. Martin-Verstraete, and are described in the study of Burguiere et al. [18]. The gene of the cystine symporter YhcL was disrupted in *B*. *subtilis* 1534 [18], while in *B*. *subtilis* 1389, the ABC binding cassette cystine transporters YtmJKLMN and YckKJI were knocked out by deletion of key genes [18]. In order to measure Hg(II) uptake in these strains as the amount converted to volatile Hg(0) (see Mercury Assay, below), we transformed the plasmid pHYRIAX with the gene (*merA*) that codes for mercury reductase (MerA) into both the wildtype and mutant strains. Transformation of the plasmid pHYRIAX (which is the shuttle vector pHY300PLK manufactured by Takara) into the three strains was accomplished by electroporation (Biorad Gene Pulser) according to the manufacturer's instructions. Transformant selection was carried out using tetracycline for *B*. *subtilis* 168, and tetracycline and spectinomycin for *B*. *subtilis* 1534. Colony PCR reactions were performed to verify that the plasmid was indeed transformed into the strains. The *Bacillus* strains containing the plasmid pHYRIAX were designated *B*. *subtilis* 168X, *B*. *subtilis* 1534X and *B*. *subtilis* 1389X. All strains used in this study are listed in Table 1.

### Mercury Assay

Mercury uptake by bacteria was quantified as the amount of added Hg(II) converted to volatile Hg(0). The cells were collected by centrifugation (4000 rpm) and washed three times with M9 minimal medium. The cultures were adjusted to an optical density (660 nm) of 0.1 in M9 defined medium. Deionized water and Hg(II) were added to acid washed 40 mL glass tubes with Teflon caps. Cysteine was prepared just before use in deionized water that had been deoxygenated by bubbling with ultrapure nitrogen. Cysteine and Hg(II) were added to the glass vials followed by 2 mL of cell culture to a final volume of 2.5 mL. In the experiment involving *Bacillus* strains, the final concentration of Hg(II) was 50 nM while the concentrations of cysteine were either 100 nM or 10 μM. The cell suspensions were left on the laboratory bench-top at room temperature without shaking, under ambient laboratory fluorescent lights, and at specific time points (0.5, 1, 2, and 3 hr) elemental mercury was removed by purging vials with ultrapure nitrogen. To each vial, 1mL of bromine monochloride (0.2 N) was then added to kill cells and liberate the remaining mercury from any complexes in solution and from any Hg still associated with the bacteria. After 24 hr of digestion with bromine monochloride, the mercury content in each vial was determined. Uptake of Hg(II) into cells was estimated by subtracting the amount of mercury remaining in each vial after purging with nitrogen from what was added and is reported as %Hg loss. Experiments with *E*. *coli* strains were performed in a similar manner with the exception that the final concentrations of Hg(II) was 10 nM while those of thiols were 100 nM for cysteine and either 100 nM and 250 nM for glutathione. All experiments were done in triplicate and the standard error is reported in each case. Statistical analysis was performed by using a t-test (P < 0.05) to compare means at equivalent time points.

### Mass balance analysis

**Fig 2:**
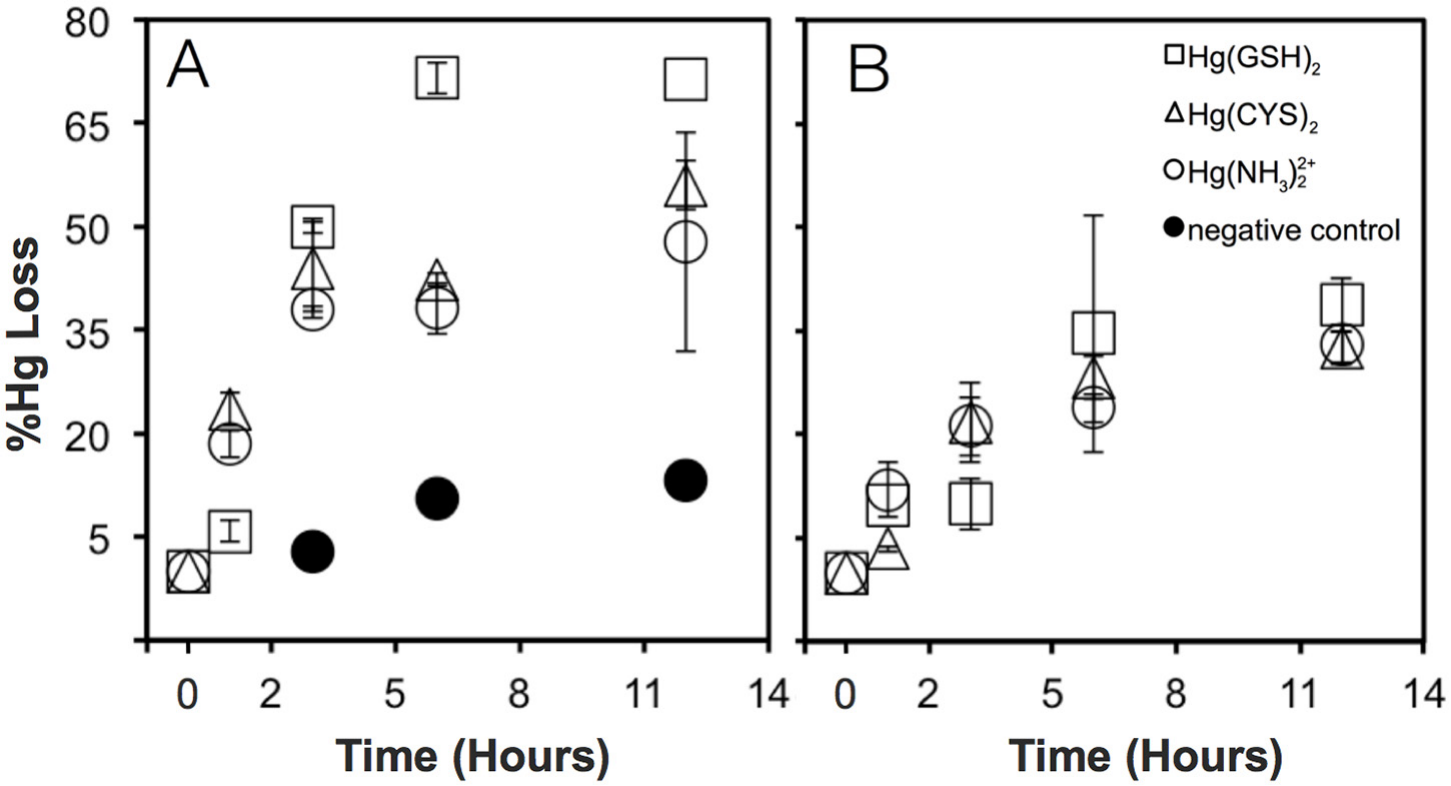
The effect of Mer transporters on the reduction of several species of Hg(II) such as Hg(NH_3_)22+(○), Hg-glutathione complex Hg(GSH)_2_ (□), and Hg-cysteine complex Hg(CYS)_2_ (Δ). Hg uptake was measured in (A) an *E*. *coli* strain (MER strain) with a functioning Mer transport system and (B) *E*. *coli* strain NRBZ1 which lacks a functioning Mer transport system. Cells were exposed to 10 nM Hg(II) with no thiol or with 100 nM glutathione or cysteine in M9 minimal media. The symbol “●” represents the negative control which is an *E*. *coli* strain without any component of the *mer* operon.

To examine the fate of Hg during these assays, a mass balance of the Hg remaining in the suspension after removal of Hg(0) by bubbling with ultra-pure N_2_ was performed at the six hour time point (Fig 2A). This experiment was performed by measuring the Hg(II) remaining after removal of Hg(0) with N_2_ and subtracting from this value the amount of Hg(II) in the liquid phase. Thus, after bubbling with N_2_, a volume of 0.500 mL of the suspension was taken for total Hg analysis, and a volume of 1.000 mL was taken for liquid phase analysis. The amount of Hg(II) in the liquid phase was obtained by centrifuging the 1.000 mL of the cell suspension, and analyzing the Hg(II) content in 0.500 mL of supernatant.

## Results and Discussion

### Bioavailability of Hg-thiol complexes in *E*. *coli*

Uptake rates of Hg(II) as cysteine or glutathione complexes were identical to those for charged inorganic complexes of Hg(II) (primarily Hg(NH_3_)_2_^2+^) in both strains of *E*. *coli* under the experimental conditions (Fig 2) according to statistical analysis (ANOVA). Since Hg(II) present in weak inorganic complexes is readily bioavailable for uptake and other reactions [9,25], identical uptake kinetics for Hg(II) for thiol complexes demonstrates that thiol-bound Hg(II) is similarly bioavailable to this bacterium. Note that in our experiments, with a Hg(II) concentration of 10 nM and a glutathione or cysteine concentration of 100 nM, nearly 100% of total Hg(II) in solution was bound by thiol according equilibrium speciation calculations made with the Mineql+ program (V.4.6). A log K of 42 [26] was used for the binding constant of Hg^2+^ with thiols in the Mineql model. Although slower than in the strain with Mer transporters (see below), uptake rates of Hg(II) from cysteine and glutathione complexes in the strain of *E*. *coli* lacking Mer transport proteins were the same as that for the uptake of Hg(II) from inorganic complexes (Fig 2B). Thus, even in the absence of Mer transporters, thiol complexes of Hg(II) are bioavailable in *E*. *coli* [11].

### The Role of Mer, Glutathione and Cystine Transporters

The effect of Mer transporters (E & T) acting in conjunction with periplasmic protein P on the uptake of thiol-bound Hg was also investigated. Reduction rates of thiol-bound Hg in the *E*. *coli* strain with an intact and functioning Mer transport system (MER strain) were compared to an *E*. *coli* strain where the genes *E*, *T* and *P* were deleted (NRBZ1). As indicated above, the uptake of both inorganic and thiol complexes of Hg(II) was faster in the *E*. *coli* strain with an intact Mer transport system than in the strain where the genes for Mer transport proteins had been knocked out (Fig 2A and 2B). The finding that Mer T & E are Hg(II) transporters is consistent with the conclusions of several other studies [22,27–29]. In addition, our results show that Mer transporters facilitate the uptake of Hg(II) regardless of whether it is present as an inorganic complex or bound to a thiol. Thus, organisms that possess the *mer* operon have greater abilities to assimilate thiol-complexes of Hg(II) than non-resistant microorganisms. How Mer transporters facilitate the uptake of thiol-bound Hg(II) is not clear. While our results do not disqualify a mechanism of uptake that involves transporting the entire Hg-thiol complex into a cell by the Mer transport system, it is more likely that Hg-thiol complexes deliver Hg efficiently to Mer transporters which then bring Hg(II) into the cell [14,19].

**Fig 3:**
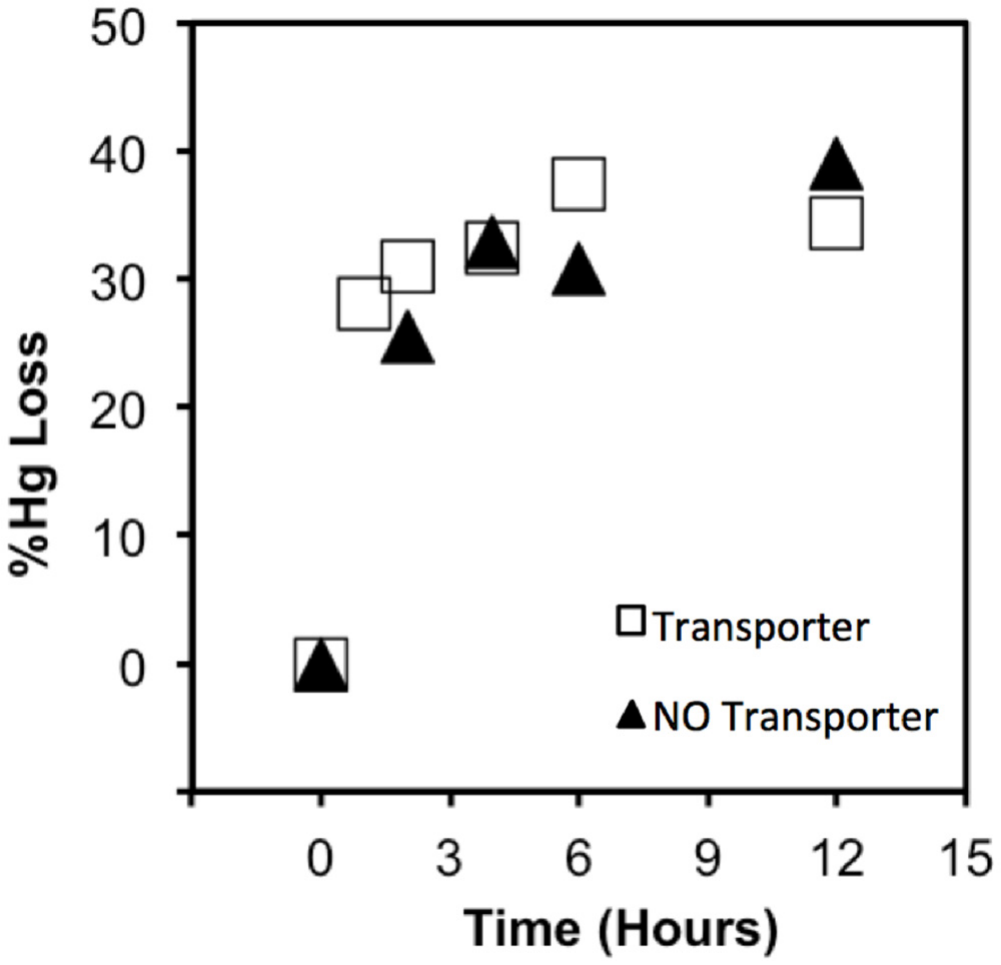
The effect of glutathione transporters on the uptake of Hg-glutathione complexes in *E*. *coli* strains with (□) and without (▲) a glutathione transport system. Cells were exposed to 10 nM Hg(II) in equilibrium with 250 nM glutathione.

Our results with a strain of *E*. *coli* lacking Mer transporters show that Hg(II) was no more bioavailable when complexed by glutathione than when bound to cysteine or ammonia (Fig 2B). This indicates that, if the uptake of glutathione complexed Hg(II) does actually occur via glutathione transporters, it is no faster than other pathways of Hg(II) uptake. To specifically test the hypothesis that the glutathione transport system is involved in the transport of Hg(GSH)_2_ into cells, uptake rates of Hg(II) from Hg(GSH)_2_ were measured in an *E*. *coli* strain (NRBZ1) with a functioning glutathione transport system and a strain (SINM35) where this transport system was disabled by knocking out two key genes (Table 1). Note that both of these strains lacked Mer transporters. In these experiments with 10 nM Hg(II) and 250 nM glutathione, 100% of Hg(II) was present as the species Hg(GSH)_2_. No difference in Hg(II) uptake rates was observed for strains with or without a functioning glutathione transport system (Fig 3). Hence, Hg(GSH)_2_ does not appear to be transported by the glutathione transport system in *E*. *coli*, rather it is likely that Hg(GSH)_2_ delivers Hg to an unidentified metal transporter on the cell membrane which then brings Hg(II) into the cell [14].

**Fig 4:**
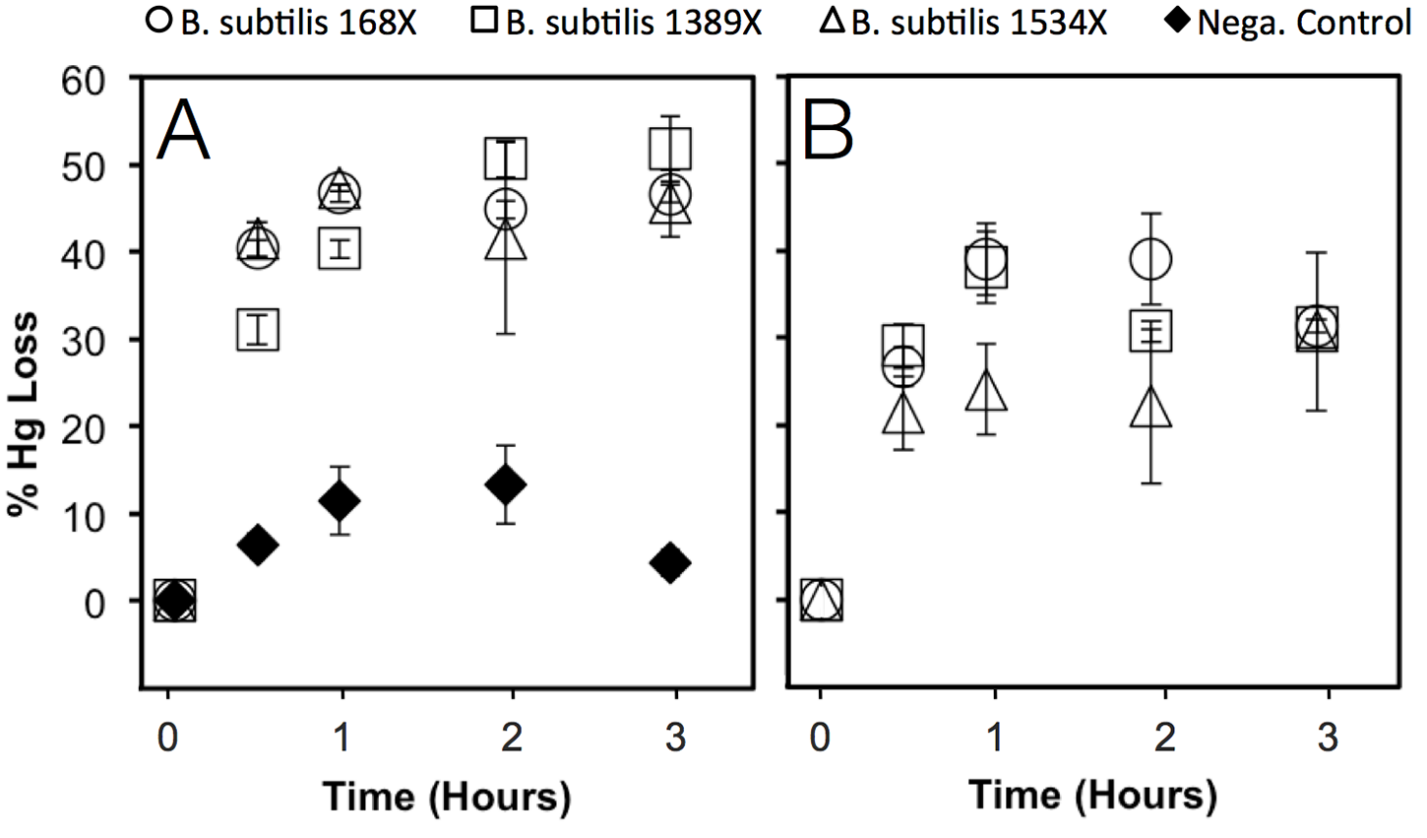
Uptake of Hg(II) (reported as percent initial Hg(II) converted to purgeable Hg(0)) in the absence (A) or presence (B) of 100 nM cysteine by *mer*A-transformed strains. The strains are *B*. *subtilis* 168X (○, wildtype), *B*. *subtilis* 1389X (□, strain without the cystine ATP binding cassette transport systems), *B*. *subtilis* 1534X (Δ, strain without cystine symporter) and *B*. *subtilis* 168 (♦, wildtype without *merA* used as a negative control). Triplicate cell suspensions (O.D. = 0.1) were exposed to 50 nM Hg(II) in M9 minimal medium for 0.5, 1, 2 and 3 hours.

We also investigated the feasibility of uptake of Hg(CYS)_2_ through cystine transporters in *B*. *subtilis* by comparing the uptake rates in the wildtype which has 3 functioning cystine transporters to mutants where one or more of these transporters has been knocked out. In order to accomplish this goal we transformed the plasmid pHYRIAX which contains the gene *merA* into the *Bacillus* wildtype and mutant strains, as detailed in the Methods. In order to confirm that MerA was active in transformed *Bacillus* strains (positive control), the reduction of Hg(II) was measured in each strain exposed to 50 nM Hg(II) in minimal medium without cysteine (Fig 4A). In addition, we conducted a negative control experiment where the wildtype strain without the plasmid was also exposed to 50 nM Hg(II). Rates of reduction of Hg(II) were rapid and approximately the same for all transformed strains in M9 minimal medium in which the dominant form of Hg(II) was Hg(NH_3_)_2_^2+^ according to MINEQL speciation analysis. Reduction of Hg(II) in the MerA-containing strains was also significantly higher (p < 0.05) than the negative control at all time points confirming that reduction in the transformed strains represents uptake and intracellular reduction. Further statistical testing using Analysis of Variance (ANOVA) indicated that the 4 data sets in Fig 4A are significantly different. The negative control represents any intra- or extracellular reduction of Hg(II) that is not related to the activity of MerA.

**Fig 5:**
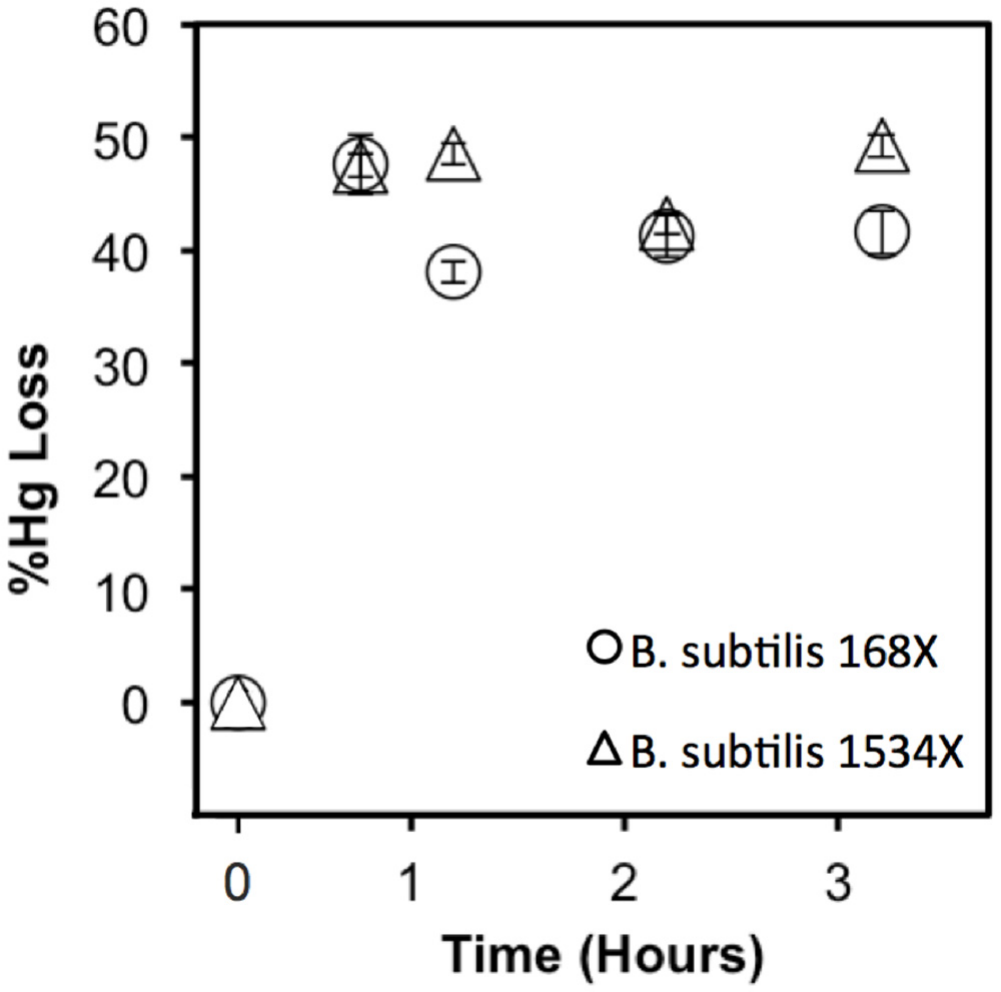
Uptake of Hg(II) (reported as percent initial Hg(II) converted to purgeable Hg(0)) in the presence of 10 μM cysteine by *mer*A-transformed strains. The strains are *B*. *subtilis* 168X (○, wildtype), and *B*. *subtilis* 1534X (Δ, strain without cystine symporter). Triplicate cell suspensions (O.D. = 0.1) were exposed to 50 nM Hg(II) in M9 minimal medium for 0.5, 1, 2 and 3 hours.

We compared uptake rates of Hg in the presence of 100 nM cysteine in the wildtype and mutants (Fig 4B). It was observed that there was no difference in uptake rates between the wildtype and mutants in which the two cystine ATP binding cassette transport systems (YtmJKLMN and YckKJI) were disrupted (B. *subtilis* 1389) indicating that there is no transport of Hg(II) through these pathways. The results of Hg(II) uptake experiments with a mutant where the cystine symporter gene was deleted were not definitive as uptake at some time points (1 hr and 2 hr) showed statistically significant reductions in uptake compared with the wildtype while other time points showed no difference. To further examine this issue, the experiment was repeated with 10 μM cysteine (Fig 5). Although uptake rates of Hg(II) in both strains in these experiments were higher than those with 100 nM cysteine, uptake rates were similar in both the wildtype and cystine symporter deletion mutant. Hence, uptake of Hg(CYS)_2_ through any of the cystine transporters in *B*. *subtilis* is doubtful. While all the results above do not validate the membrane exchange hypothesis, they certainly cast doubt on the thiol-transporter hypothesis as a valid mechanism of Hg uptake in bacteria. A good follow-up experiment that might help validate the membrane exchange hypothesis would be to compare uptake rates of Hg(II) from Hg-thiol complexes in a strain with a functioning MerP to a strain where the gene governing this protein has been knocked out. If a higher uptake rate is observed in the strain with a functioning MerP then that would be evidence supporting the validity of the membrane-exchange hypothesis.

### Evaluation of the Biosensor Approach

In our biosensor, the intracellular reduction of Hg(II) by MerA provides a quantitative estimate of Hg(II) bioavailability. In prior studies short-term microbial uptake/bioavailability of Hg has often been quantified as the difference between Hg(II) associated with the cell (both that bound to the cell surface plus the amount remaining in solution) and the initial amount of Hg(II) added [13,14,30,31]. Various cell surface “washing” procedures prior to analysis have been used to remove surface bound Hg [13,30]. The intracellular, enzyme-catalyzed reduction of Hg(II) in our biosensor approach has the advantage of not requiring a cell-surface wash to correct for adsorbed or non-specifically surface bound Hg. As the enzyme MerA is located inside the cell, the amount of Hg(II) reduced can serve as a surrogate for Hg(II) uptake as demonstrated in this study. In addition, with this technique short-term Hg uptake kinetics can be tracked in real time.

To examine the fate of Hg during these assays, a mass balance of the Hg remaining in the suspension after removal of Hg(0) by bubbling with ultra-pure N_2_ was performed as described in the methods. The results demonstrated that most of the Hg(II) remaining was in the liquid phase (72%), and a smaller percentage (28%) was associated with cells. The results indicate that there is Hg(II) associated with cells that is not bioavailable to the internal Mer enzymes. It is very likely that this Hg(II) is associated with cell surface or internal membranes and is out of the reach of the Hg-reducing enzymes. Bioavailability is therefore distinguished by our assays from total uptake (i.e the total amount of Hg associated with cells) as the assay determines the Hg(II) that is associated with cells and also available for metabolic processing by the cell.

In interpreting the assay, it is necessary to consider other processes that could reduce Hg(II) or oxidize Hg(0) within the medium or at the surface/within cells as such processes would influence the net amount of Hg(0) in the medium. If net oxidation were to occur in the medium, then the measurement of Hg(0) would be an underestimate of the uptake into the cells. For the negative controls (see Figs 2 and 4), which are suspensions of cells without MerA, there is some reduction occurring, with a maximum amount of 10–15%. This is much less than the values found in the other assays, suggesting that most of the Hg(0) formed is via the MerA system. Past research has shown that Hg(0) can be readily oxidized to Hg(II) in the presence of thiols [32–35]. The rate of oxidation is greatly enhanced under anoxic conditions with rates equivalent to oxidation of 70–90% of the initial Hg(0) within 48 hours [33]. However, under abiotic oxic conditions, the rate of oxidation of Hg(0) in the presence of cysteine was calculated to be about 0.23% after 10 hours [35], or 0.02%/hr which would make such processes insignificant under the conditions of our experiments. In addition, Smith et al [36] concluded that although cells can oxidize Hg(0) to Hg(II), the rate of oxidation is much slower than the rate of reduction mediated by the enzyme MerA [36]. For all these reasons, oxidation of Hg(0) is not expected to interfere with the results of the experiments, and additionally the reduction measured is primarily due to Mer-associated reduction.

### Environmental Significance

Understanding the microbial accumulation of Hg from Hg-thiol complexes is critically important to understanding Hg transformations in aquatic environments and the accumulation of Hg in aquatic food webs. Glutathione and cysteine are two of the most abundant thiols found in surface waters [37–40] and are also abundant in sediment pore waters where they may be present at nanomolar to micromolar concentrations depending on season and depth of sediments [41]. Because glutathione and cysteine have high binding constants with Hg^2+^ (Log K ≈ 42 for Hg(RSH)_2_ complexes)[26], when present in concentrations above 2 nM, thermodynamic models indicate that a major fraction (35 to 80%) of dissolved Hg(II) in coastal waters will be bound to these thiols (data not shown). Results from our experiments using a microbial biosensor in which Hg uptake is quantified as the intracellular reduction of Hg(II) to Hg(0) by mercuric reductase (MerA) suggest that Hg(II) bound to cysteine and glutathione is just as bioavailable to bacteria as Hg(II) bound to inorganic complexes. This suggests that the rate limiting step in Hg(II) uptake in the presence of inorganic and small molecular weight organic ligands is internalization of the Hg across the membrane and that the speciation in solution does not exert a strong influence on the uptake rate. This contrasts the situation where Hg is bound to humic acids and other larger molecular weight ligands where the uptake rate depends on the degree of complexation of the Hg to these ligands [11,42]. In this case, large molecular weight ligands slow the exchange of Hg between the complex in solution and the surface binding site, and therefore reduce the Hg uptake rate. Using our MerA biosensor, we also show that Hg transport proteins from Hg-resistant bacteria (i.e. Mer transporters) enhance the uptake of Hg(II) from inorganic and thiol complexes while the glutathione and cystine transport systems in bacteria lacking Mer transporters do not facilitate uptake of glutathione or cysteine-bound Hg, respectively. Thus the transport of Hg(II) from aqueous thiol complexes into bacterial cells lacking Hg resistance genes remains to be described.

Understanding Hg accumulation in Hg-resistant bacteria is important since they can play a significant role in the biogeochemical cycling of Hg in terrestrial and aquatic environments [19]. For example, it was demonstrated that several bacteria in a coastal lagoon in the High Arctic possessed and expressed the *merA* gene and it was estimated that Hg-resistant bacteria were responsible for as much as 90% of Hg(0) formed in this environment [43]. Since Hg(0) is volatile, it is easily lost to the atmosphere and accounts for most of the flux of Hg from the ocean to the atmosphere [44]. The accumulation of Hg from thiol complexes by various microbes and its link to Hg reduction in marine and freshwater systems is therefore of critical importance to Hg cycling and needs further study. We point out that the experiments performed in this study were carried out under aerobic conditions and do not apply to anoxic sediments where methylation rates are highest.
